# Factors related to condomless anal intercourse between men who have sex with men: results from a European bio-behavioural survey

**DOI:** 10.1093/pubmed/fdz052

**Published:** 2019-05-15

**Authors:** N S Sherriff, A M Jones, M Mirandola, L Gios, U Marcus, C Llewellyn, M Rosinska, C Folch, S Dias, I Toskin, I Alexiev, S Kühlmann-Berenzon, Sialon II Network, Massimo Mirandola, Christiana Nöstlinger, Ivailo Alexiev, Ulrich Marcus, Barbara Suligoi, Saulius Caplinskas, Magdalena Rosińska, Sónia Ferreira Dias, Alexandru Rafila, Danica Staneková, Irena Klavs, Cinta Folch, Inga Velicko, Igor Toskin, Nigel Sherriff

**Affiliations:** 1 School of Health Sciences, University of Brighton, Brighton, BN1 9PH, UK; 2 Centre for Transforming Sexuality & Gender, University of Brighton, Brighton, Brighton, BN1 9PH, UK; 3 Research and Development Department, Sussex Partnership NHS Foundation Trust, Worthing, United Kingdom, BN13 3EP, UK; 4 Infectious Diseases Section, Department of Diagnostics and Public Health, University of Verona, Verona, Italy; 5 Department of Infectious Disease Epidemiology, Robert Koch Institute, Berlin, Germany; 6 Brighton and Sussex Medical School, University of Sussex, Brighton, BN1 9PH, UK; 7 National Institute of Public Health-National Institute of Hygiene, Warsaw, Poland; 8 Centre d’Estudis Epidemiològics sobre les Infeccions de Transmissió Sexual i Sida de Catalunya (CEEISCAT), Dept Salut, Generalitat de Catalunya / CIBER Epidemiologia y Salud Pública (CIBERESP), Barcelona, Spain; 9 Escola Nacional de Saúde Pública, Centro de Investigação em Saúde Pública & GHTM, Universidade NOVA de Lisboa, Portugal; 10 Department of Reproductive Health and Research, World Health Organization, Geneva, Switzerland; 11 National Reference Laboratory of HIV, National Centre of Infectious and Parasitic Diseases, Sofia, Bulgaria; 12 Department of Public Health Analysis and Data Management, Public Health Agency of Sweden, Solna, Sweden; 13 Sialon II Network (Short List)

**Keywords:** HIV, MSM, relationships, respondent-driven sampling, time-location sampling

## Abstract

**Background:**

Relationship status is an important factor associated with condomless anal intercourse (CAI) amongst men who have sex with men (MSM).

**Methods:**

A multi-centre bio-behavioural survey with MSM was conducted in 13 European cities (*n* = 4901) exploring factors associated with CAI via bivariate and multivariate multilevel logistic regression analyses.

**Results:**

Likelihood of CAI with casual partners was associated with being ‘out’ to a majority (AOR = 1.19;95% CI 1,1.42); knowing their HIV status (AOR = 1.86; 95% CI 1.25,2.76); using substances (1–2 AOR = 1.39; 95% CI 1.16,1.63, 2+ AOR = 1.81; 95% CI 1.35,2.42); being older (AOR = 0.98; 95% CI 0.97,0.99); successful sero-communication (AOR = 0.79; 95% CI 0.67,0.94); and, not having a recent HIV test (AOR = 0.78; 95% CI 0.66,0.92). CAI with steady partners was associated with successful sero-communication (AOR = 2.72; 95% CI 2.72,3.66); not having a recent HIV test (AOR = 1.26; 95% CI 1.09,1.46), and; being older (AOR = 0.99; 95% CI 0.98,0.99).

**Conclusions:**

Understandings of partner type and/or relationship status in relation to CAI amongst MSM can potentially play an important role in the development of culturally appropriate HIV/STI prevention and risk-reduction efforts targeting at-risk MSM. Our results speak to the need to consider segmented and tailored public health and health promotion initiatives for MSM with differing CAI behaviours and relationship profiles.

## Introduction

Epidemiological evidence suggests that sex between men continues to be the main mode of HIV transmission accounting for 40% of all new diagnoses in 2016 across the European Union (EU) and the European Economic Area (EEA).^1^ However, although there is now evidence of decreasing diagnoses amongst men who have sex with men (MSM) in some countries including Austria, Belgium, Italy, the Netherlands, Spain, and the United Kingdom,^2^ in other EU/EEA countries diagnoses have increased substantially.^1^ Such distinct trends mean that it is essential to sustain and, in some cases, strengthen HIV prevention interventions tailored to the local epidemiological context and targeting population groups most at risk; for many countries this means MSM.

In order to develop and implement community-level risk-reduction initiatives targeting MSM, it is necessary to examine not only key sexual behaviours amongst different MSM (sub) populations, but to also understand and consider the context in which they occur; relationships are one such context. Indeed, research demonstrates that relationship status and/or partnership type is an important factor associated with condomless anal intercourse (CAI) and subsequent risk for HIV and sexually transmitted infections (STIs).^3–7^

However, the risk for HIV and other STI acquisition is dependent on other factors than just CAI. Kramer and colleagues have drawn attention to this issue and note that although prevention initiatives commonly target individualistic-behaviours thus regarding CAI as an inherently ‘risky’ sexual behaviour, such approaches can be unhelpful as they may mask more complex and dynamic issues occurring within MSM in both steady and casual or non-steady relationships including the use of risk-reduction strategies.^8–10^ For instance the number of partners as well as knowledge of own and partner’s HIV serostatus, the use of ‘negotiated safety’ agreements,^11^ serosorting, and the effective use of anti-retroviral drugs to lower viral load as well as the use of pre-exposure prophylaxis (PreP)^12,13^ may be contingent on the perception of the type of relations.

Nevertheless, examining explanatory factors related to differences in CAI between MSM in steady and casual relationships can be important in order to interrogate segmented public health and health promotion initiatives for MSM with differing sexual behaviour and relationship profiles. Consequently, in this present analysis we utilize data from the EU-funded Sialon II study which was a large multi-centre biological and behavioural cross-sectional survey of MSM in community settings carried out across 13 European cities. The objectives of our analysis were to: (i) investigate CAI and explanatory variables amongst MSM in a large community sample; (ii) explore the differences in CAI between those participants who had steady partners with those who had casual or non-steady partners, and finally; (iii) potentially inform the development (and assist implementation) of risk-reduction initiatives targeting MSM.

## Methods

### Study design

Detailed study methods are reported elsewhere.^14–16^ In summary, the Sialon II study was a complex multi-centre integrated bio-behavioural cross-sectional survey with a concomitant collection of behavioural data and biological data (oral fluid or blood specimens).

### Setting

The survey was implemented in 13 European cities. The decision to use Time-Location Sampling (TLS) or Respondent Driven Sampling (RDS) to recruit participants was based on preliminary formative research. TLS (also known as Venue Day Time Sampling, Temporal Spatial Sampling, and Time Venue Sampling) was used to recruit participants in Brussels, Sofia, Hamburg, Warsaw, Lisbon, Ljubljana, Barcelona, Stockholm, and Brighton (*n* = 3596). TLS is a quasi-probabilistic method used to recruit members of a target population at specific times in set venues.^17^ In this study, the venues or settings for data collection included social and/or commercial venues and cruising settings preliminarily identified through formative research and which were then selected randomly for data collection sampling calendars.^18^ RDS was used in Bratislava, Bucharest, Verona, and Vilnius (*n* = 1305). RDS is similar to snowball sampling in that it requires the target population to be socially networked so participants can invite their peers to participate. However, RDS is different in that it incorporates numerous theoretical assumptions to reduce the numerous biases found in standard snowball sampling methods (see^19^). Enrolment for RDS in Sialon II was based on the individuals’ social network and for the data collection, locally accredited healthcare facilities (e.g. a hospital) were used. In TLS cities, participants were recruited during 2013, whilst in RDS cities recruitment started in 2013 and finished in 2014. Prior to the survey we estimated a 50% response rate as part of the sample size calculations. A data collection procedure to record refusals was therefore developed for TLS only. However, not all sites collected this data (with exception of the Brighton site with a 59% response rate). Thus an overall response and/or refusal rate for the TLS survey is not reported.

### Participants

Participants were men present in the cities at the moment of data collection (2013–14) who met the inclusion criteria (18 years or older; had sex with another man during the previous 12 months, and; agreed to donate an oral fluid or blood specimen depending on the sampling approach adopted). Exclusion criteria were being younger than the legal age of consent (18 years old) or having already participated in the study.

### Instruments

A self-administered pen-and-paper questionnaire was used to collect behavioural data. The preliminary version of the questionnaire was designed by the Sialon II network in line with the Global AIDS Monitoring indicators (GAM)^16,20^ and previous EC-funded European projects (e.g.^21,22^) and then piloted amongst MSM in each study site. The English version of the questionnaire was translated into local languages and back-translated into English.

### Ethics

Research protocols were submitted to, and approved by, an institutional ethical review board in each participating city, as well as by the WHO Research Project Review Panel (RP2) and the WHO Research Ethics Review Committee (ERC). All participants were given a study information sheet and the details were read out to ensure they understood what the study involved, that participation was voluntary, and that they had the right to withdraw at any time without giving a reason. Those willing to take part then signed a consent form. For TLS and RDS, a dedicated barcode system was used in order to link anonymously the different types of data collected (i.e. biological samples and behavioural data). For the TLS survey, respondents who wanted to collect their tests results could do so using their unique bar code ID. For the RDS survey where respondents were tested directly in a hospital/clinical setting, test results were available according to the local standards (including pre and post-test counselling).

### Measures

#### Outcome variables

The primary focus of this analysis was to explore engagement in CAI measured as insertive/receptive unprotected anal intercourse in the last 6 months. Since we initially expected the ‘risk’ behaviour for those engaging in CAI in the last 6 months to differ depending on the relationship status (steady or casual partner), two separate ‘primary’ outcomes were created for two separate analyses. The first outcome indicated whether an individual had engaged in CAI with one or more (yes = 1) or zero (no = 0) steady partners. This included ‘boyfriends’ and ‘husbands’ (i.e. not being ‘single’) and excluded partners who were ‘sex buddies’. The second outcome indicated whether individuals had engaged in CAI with one or more (yes = 1) or zero (no = 0) casual partners. Casual partners were defined as: those with whom one had had sex with only once (e.g. a ‘one-night stand’); and those with whom one had sex with more than once but were not considered a steady partner (such as sex buddies). Some participants categorized current relationships as a mix of casual and steady partners since the two categorizations are not mutually exclusive.

#### Independent variables

Independent variables included: age (based on self-reported year of birth), education level (secondary school or lower, high school/post-secondary education/vocational school or college, or university degree/higher), migrant status (based on country of birth and country of residence: native, emigrant, immigrant or visitor), ‘outness’ (the extent to which participants reported being open about their sexual attraction towards men with others: being out to ‘less than half’ or ‘out to the majority’), overall perceived attitude towards gay or bisexual people at work/school and amongst parents/friends/acquaintances (positive, neutral or negative attitude), HIV testing in the last 12 months and results known (no or yes), knowledge of own HIV status (using both self-reported status and status based on laboratory results: newly diagnosed, negative test result, already known), sex role at last anal sex (insertive, receptive, versatile), number of substances (type specified in the questionnaire) used at last anal sex (0, 1–2, 2+), frequency of visits to gay venues during last 3 months where sex-on-premises is possible (0 ‘no’, 1–3 ‘low’ 3+ ‘high’), currently having sex with women (no or yes), serostatus communication at last anal intercourse (successful, unsuccessful; this constructed variable distinguishes between successful serostatus disclosure [i.e. a communication that establishes HIV serostatus concordance or discordance, including unilateral HIV infection disclosure], and unsuccessful serostatus disclosure [i.e. a communication where either none or only one of the involved partners disclosed his serostatus, with the exception of unilateral HIV infection disclosure]), see.^23^

### Data analysis

#### Descriptive analysis

For continuous variables median and interquartile range (IQR) were used. For nominal variables count and percentages were used. The Chi-square test was used to examine the relation between CAI in casual partners and CAI in steady partners as well as to compare CAI rates between pairs of cities.

#### Bivariate and multivariate multilevel modelling

For all bivariate and multivariate analyses, factors associated with CAI were identified using a two-level multilevel logistic regression model with a random intercept at the city level. The random component accounts for the hierarchical nature of the data. Analyses were carried out on all available cases.

The first step to building a model was to identify those individual independent variables (from the full list above) that were statistically significantly associated with CAI using bivariate analysis. Variables from this pool of potential risk factors were then used for inclusion in the multivariate analysis. The variables were added to the null model one by one using a forward selection process choosing the most significant (*P* < 0.05) variable first. The likelihood ratio test was used to compare the new model with the nested model. For all statistical tests, significance was indicated by *P* < 0.05. The final model estimated the adjusted odds ratios (AORs) and the corresponding 95% confidence interval (95% CI) for factors associated with CAI. We then used the resulting model to explore the relationship between age and risk of engagement in CAI for each city. Analyses were first carried out for modelling CAI with casual partners and then repeated for steady partners. Stata® Version 13 was used for all analyses (College Station, TX: StataCorp LP).

## Results

Of 4901 participants who completed the survey, 4340 (88.55%) had sex in the last 6 months and were included in the analysis. The median age was 32 years with an IQR of 15 years. 3624 (83.50%) had at least one casual partner, 2911 (67.07%) had at least one steady partner and 2195 (50.58%) had both. 1374 (31.66%) participants reported CAI with casual partners (median age 31 years; IQR 12 years) and 1482 (34.15%) with steady partners (median age 31 years; IQR 14 years) and 687 (15.83%) reported CAI with both types of partner (median age 30 years; IQR 13 years). Median age for the 2171 (50.02%) who did not have CAI with casual or steady partners was 33 years (IQR 16 years). There was also a significant association (*P* < 0.001) between participant reports of CAI with casual partners and CAI with steady partners. Those who had CAI with steady partners had 2.73 times higher odds of CAI with casual partners (odds = 0.862) compared to those who did not have CAI with steady partners (odds = 0.316).

CAI varied between cities and by relationship status (Table 1). Brussels had the lowest percentage rate of CAI with casual partners and Sofia had the highest (22.7% vs. 53.3%, respectively; *P* = 0.001). Barcelona saw the lowest percentage rate of CAI with steady partners (23.81%) whilst Vilnius (40.34%) had the highest (*P* < 0.001). Table 1 presents the main characteristics of the study population stratified by relationship status. Odds ratios from the bivariate analyses are displayed in Table 2; all statistically significant variables made up the pool of potential factors for the final model.

**Table 1:** Characteristics of study participants

Factor	Total sample		Has at least one casual partner	Percentage of total population	Has at least one steady partner	Percentage of total population
	Count	%	Count	%	Count	%
**Age**						
18–24	865	19.9	321	37.1	319	36.9
25–34	1708	39.4	596	34.9	627	36.7
35–44	989	22.8	297	30.0	320	32.4
45–54	530	12.2	111	20.9	163	30.8
55+	244	5.6	48	19.7	53	21.7
Total	4336	100	1373	31.7	1482	34.2
**Highest education level**						
Secondary or lower	251	5.9	69	27.5	60	23.9
High school or post-secondary	1599	37.5	569	35.6	550	34.4
University or higher	2413	56.6	712	29.5	847	35.1
Total	4263	100	1350	31.7	1457	34.2
**Perceived attitude towards homosexuality & bisexuality**						
Positive	1921	44.7	596	31.0	698	36.3
Neutral	1655	38.5	556	33.6	577	34.9
Negative	725	16.9	214	29.5	196	27.0
Total	4301	100	1366	31.8	1471	34.2
**Outness**						
Out to less than half	1776	41.6	558	31.4	558	31.4
Out to majority	2498	58.4	804	32.2	908	36.3
Total	4274	100	1362	31.9	1466	34.3
**HIV testing in last 12 months and test result known**						
Yes	2335	57.4	805	34.5	803	34.4
No	1733	42.6	478	27.6	581	33.5
Total	4068	100	1283	31.5	1384	34.0
**Sex role**						
Insertive	1379	36.1	439	31.8	479	34.7
Receptive	1320	34.6	487	36.9	461	34.9
Versatile	1119	29.3	343	30.7	426	38.1
Total	3818	100	1269	33.2	1366	35.8
**No. of substances used**						
No drugs	1895	44.8	515	27.2	682	36.0
1–2 drugs	1982	46.9	704	35.5	659	33.2
> 2 drugs	350	8.3	146	41.7	133	38.0
Total	4227	100	1365	32.3	1474	34.9
**HIV status/knowledge**						
Tested negative	3716	91.1	1134	30.5	1263	34.0
Newly diagnosed	146	3.6	52	35.6	46	31.5
Already known	215	5.3	95	44.2	77	35.8
Total	4077	100	1281	31.4	1386	34.0
**Had sex with female partners**						
No	3266	85.7	1050	32.1	1178	36.1
Yes	543	14.3	170	31.3	154	28.4
Total	3809	100	1220	32.0	1332	35.0
**Frequentation of sex venues**						
No (0)	1091	25.9	344	31.5	405	37.1
Low (1–3)	1772	42.1	511	28.8	589	33.2
High (3+)	1350	32.0	495	36.7	463	34.3
Total	4213	100	1350	32.0	1457	34.6
**Serostatus communication**						
Unsuccessful	2498	64.6	891	35.7	688	27.5
Successful	1369	35.4	413	30.2	720	52.6
Total	3867	100	1304	33.7	1408	36.4
**Migration Status**						
Native	3557	82.2	1159	32.6	1201	33.8
Emigrant	60	1.4	26	43.3	31	51.7
Immigrant	492	11.4	130	26.4	156	31.7
Visitor	219	5.1	56	25.6	89	40.6
Total	4328	100	1371	31.7	1477	34.1
**City**						
Barcelona	357	8.2	85	23.8	85	23.8
Bratislava	374	8.6	163	43.6	140	37.4
Brighton	354	8.2	97	27.4	132	37.3
Brussels	352	8.1	80	22.7	120	34.1
Bucharest	160	3.7	70	43.8	55	34.4
Hamburg	350	8.1	102	29.1	99	28.3
Lisbon	376	8.7	99	26.3	141	37.5
Ljubljana	346	8.0	84	24.3	134	38.7
Sofia	409	9.4	218	53.3	154	37.7
Stockholm	249	5.7	74	29.7	85	34.1
Verona	364	8.4	104	28.6	115	31.6
Vilnius	295	6.8	98	33.2	119	40.3
Warsaw	354	8.2	100	28.2	103	29.1
Total	4340	100	1374	31.7	1482	34.1

**Table 2:** Results from bivariate multilevel models identifying potential risk factors for CAI with partners by relationship status

CAI with casual partners vs no CAI with casual partners										CAI with steady partners vs no CAI with steady partners							
Independent Variables		OR	SE	*z*	*P* > *z*	95% Confidence interval for Odds ratio		Chi-square	*P*-value	OR	SE	*z*	*P* > z	95% Confidence interval for Odds ratio		Chi-square	P-value
						Lower	Upper							Lower	Upper		
**Age**								36.29	<0.001							25.33	<0.0001
		0.98	<0.01	−6.02	<0.001	0.97	0.99			0.98	<0.01	−5.03	<0.001	0.98	0.99		
	Const.	0.92	0.14	−0.57	0.57	0.68	1.23			0.89	0.11	−0.98	0.33	0.70	1.12		
City	Var(const)	0.11	0.05			0.05	0.26			0.02	0.01			0.01	0.08		
**Highest Education level**								12.86	<0.001							9.84	0.0073
	Primary	Ref								Ref							
	High school	1.40	0.22	2.16	0.031	1.03	1.90			1.60	0.25	2.94	0.003	1.17	2.18		
	University	1.10	0.17	0.64	0.522	0.82	1.49			1.63	0.26	3.13	0.002	1.20	2.22		
	const	0.38	0.07	−5.36	<0.001	0.27	0.54			0.33	0.05	−7.10	<0.001	0.24	0.45		
City	Var(const)	0.15	0.06			0.06	0.35			0.03	0.02			0.01	0.09		
**Perceived attitude towards homosexuality & bisexuality**								11.20	0.0037							25.09	<0.001
	Positive	Ref								Ref							
	Neutral	0.95	0.07	−0.65	0.513	0.82	1.10			0.89	0.06	−1.65	0.099	0.77	1.02		
	Negative	0.71	0.07	−3.27	0.001	0.58	0.87			0.60	0.06	−5.00	<0.001	0.50	0.74		
	const	0.49	0.06	−5.64	<0.001	0.39	0.63			0.59	0.04	−7.02	<0.001	0.51	0.68		
City	Var(const)	0.17	0.07			0.07	0.39			0.04	0.02			0.02	0.12		
**Outness**								15.85	0.0001							16.70	<0.001
	Out to less than half	Ref								Ref							
	Out to majority	1.34	0.10	3.98	<0.001	1.16	1.55			1.33	0.09	4.09	<0.001	1.16	1.53		
	const	0.39	0.05	−7.25	<0.001	0.30	0.50			0.44	0.03	−10.51	<0.001	0.38	0.51		
City	Var(const)	0.18	0.08			0.08	0.42			0.04	0.02			0.02	0.12		
**HIV testing in last 12 months and result known**								19.80	<0.001							0.45	0. 5044
	Yes	Ref								Ref							
	No	0.73	0.05	−4.45	<0.001	0.63	0.84			0.96	0.07	−0.67	0.504	0.84	1.09		
	const	0.52	0.06	−5.40	<0.001	0.41	0.66			0.52	0.03	−9.76	<0.001	0.46	0.60		
City	Var(const)	0.16	0.07			0.07	0.38			0.03	0.02			0.01	0.10		
**Sex role**								6.98	0.0305							4.20	0.1222
	Insertive	Ref								Ref							
	Receptive	1.19	0.10	2.08	0.037	1.01	1.40			1.00	0.08	−0.04	0.968	0.85	1.17		
	Versatile	0.96	0.09	−0.46	0.647	0.81	1.14			1.16	0.10	1.79	0.073	0.99	1.37		
	const	0.47	0.05	−6.53	<0.001	0.37	0.59			0.53	0.04	−7.96	<0.001	0.46	0.62		
City	Var(const)	0.13	0.06			0.05	ß0.31			0.04	0.02			0.01	0.11		
**Substances used**								56.72	<0.001							5.79	0.0552
	None	Ref								Ref							
	1–2	1.48	0.11	5.40	<0.001	1.28	1.70			0.87	0.06	−1.97	0.049	0.76	1.00		
	>2	2.35	0.30	6.76	<0.001	1.84	3.02			1.10	0.14	0.75	0.456	0.86	1.40		
	const	0.36	0.04	−8.34	<0.001	0.28	0.46			0.56	0.04	−8.23	<0.001	0.49	0.65		
City	Var(const)	0.16	0.07			0.07	0.36			0.03	0.02			0.01	0.10		
**HIV status knowledge**								31.32	<0.001							0.5931	0.5931
	Tested negative	Ref								Ref							
	Newly diagnosed	1.33	0.24	1.56	0.119	0.93	1.89			0.91	0.17	−0.51	0.609	0.64	1.30		
	Already known	2.21	0.32	5.46	<0.001	1.67	2.95			1.14	0.17	0.86	0.391	0.85	1.52		
	const	0.43	0.05	−7.56	<0.001	0.34	0.53			0.51	0.03	−11.2	<0.001	0.46	0.58		
City	Var(const)	0.15	0.06			0.06	0.34			0.03	0.02			0.01	0.09		
**Had sex with female**								2.63	0.1047							13.22	0.0003
	No	Ref								Ref							
	Yes	0.84	0.09	−1.62	0.105	0.69	1.04			0.68	0.07	−3.64	<0.001	0.56	0.84		
	const	0.47	0.06	−6.13	<0.001	0.37	0.60			0.56	0.04	−8.60	<0.001	0.49	0.64		
City	Var(const)	0.18	0.08			0.08	0.41			0.04	0.02			0.01	0.12		
**Venues frequency**								7.71	0.0211							2.53	0.2826
	No(0)	Ref								Ref							
	Low (1–3)	1.00	0.09	0.04	0.97	0.84	1.19			0.89	0.07	−1.42	0.155	0.75	1.05		
	High (3+)	1.27	0.13	2.27	0.023	1.03	1.57			0.87	0.09	−1.40	0.163	0.72	1.06		
	const	0.43	0.05	−6.78	<0.001	0.34	0.55			0.58	0.05	−6.70	<0.001	0.49	0.68		
City	Var(const)	0.14	0.06			0.06	0.32			0.03	0.02			0.01	0.09		
**Serostatus communication**								6.56	0.0104							235.77	<0.001
	Unsuccessful	Ref								Ref							
	Successful	0.83	0.06	−2.56	0.01	0.72	0.96			2.99	0.21	15.35	<0.001	2.60	3.44		
	const	0.53	0.06	−5.88	<0.001	0.43	0.66			0.38	0.03	−12.88	<0.001	0.33	0.44		
City	Var(const)	0.12	0.05			0.05	0.30			0.05	0.03			0.02	0.13		
**Migration Status**								3.27	0.3514							11.98	0.0075
	Native	Ref								Ref							
	Emigrant	1.57	0.43	1.67	0.095	0.92	2.68			2.05	0.54	2.73	0.006	1.22	3.42		
	Immigrant	0.94	0.11	−0.58	0.564	0.75	1.17			0.96	0.10	−0.34	0.732	0.78	1.19		
	Visitor	0.96	0.16	−0.26	0.794	0.68	1.34			1.37	0.21	2.11	0.035	1.02	1.85		
	const	0.46	0.05	−7.1	<0.001	0.37	0.57			0.50	0.03	−11.29	<0.001	0.45	0.57		
City	Var(const)	0.14	0.06			0.06	0.33			0.03	0.02			0.01	0.09		

^*Notes*: *z* = test statistic for an individual category in the bivariate model; *P* > *z* = significance of an individual category in the bivariate model; Wald Chi-square statistic and *P*-value are used to test the significance of a whole variable in the bivariate model; SE = Standard Error; OR: Odds Ratio^

### Casual partners

The results from the multivariate analyses are shown in Table 3 (casual partner). The analysis showed that CAI with casual partners was more likely amongst those who were ‘out’ to a majority (AOR = 1.19; 95% CI 1,1.42, *P* = 0.047); who knew their HIV status (AOR = 1.86; 95% CI 1.25,2.76, *P* = 0.002); who used 1–2 substances (drugs/alcohol; AOR = 1.39; 95% CI 1.16,1.63, *P* < 0.001); and, who used two or more substances (AOR = 1.81; 95% CI 1.35,2.42, *P* < 0.001). Being older (AOR = 0.98; 95% CI 0.97,0.99, *P* < 0.001); having successful sero-communication (AOR = 0.79; 95% CI 0.67,0.94, *P* = 0.006); and, not having had a recent HIV test (AOR = 0.78; 95% CI 0.66,0.92, *P* = 0.002), were all associated with reductions in the likelihood of CAI.

**Table 3:** Multilevel model results identifying risk factors for CAI with casual partners compared to no CAI with casual partners

Risk factor	Category	AOR	SE	95% Confidence Interval		*P*-value
				Lower	Upper	
**Outness**	Out to less than half	Ref				
	Out to majority	1.19	0.11	1.00	1.42	0.047
**Had HIV test in last 12 months and results known**	Yes	Ref				
	No	0.78	0.07	0.66	0.92	0.002
**Sex role**	Insertive	Ref				
	Receptive	1.18	0.11	0.98	1.41	0.082
	Versatile	0.88	0.09	0.72	1.07	0.174
**Serostatus communication**	Unsuccessful					
	Successful	0.79	0.07	0.67	0.94	0.006
**Highest Educational level**	Secondary or lower	Ref				
	High school	1.05	0.20	0.73	1.54	0.811
	University	0.85	0.16	0.59	1.22	0.375
**Age**						
	Continuous	0.98	<0.01	0.97	0.99	<0.001
**HIV status knowledge**	Tested negative	Ref				
	Newly diagnosed	1.04	0.22	0.68	1.56	0.851
	Already known	1.86	0.37	1.25	2.76	0.002
**Substances used**	None	Ref				
	1–2 drugs	1.39	0.12	1.16	1.63	<0.001
	>2 drugs	1.81	0.27	1.35	2.42	<0.001
Constant		0.89	0.25	0.52	1.53	0.067
City	Variance (Constant)	0.13	0.06		0.05	0.32

LR test vs. logistic regression: chibar2(01) = 47.57 Prob. ≥ chibar2 = 0.0000

*Notes:* Adjusted Odds Ratio (AOR); Standard Error (SE)

### Steady partners

With reference to the multivariate analyses in Table 4 (steady partner), CAI with a steady partner was more likely for those with successful sero-communication (AOR = 2.72; 95% CI 2.72,3.66, *P* < 0.001) and for those who had not been tested for HIV in the last 12 months (AOR = 1.26; 95% CI 1.09,1.46, *P* = 0.002). It was also approaching significance for those who reported being out to a majority (AOR = 1.16; 95% CI 1.00,1.36, *P* = 0.054). Reduced likelihood of CAI with a steady partner was associated with increasing age for all cities (AOR = 0.99; 95% CI 0.98,0.99, p,0.001).

**Table 4:** Multilevel model results identifying risk factors for CAI with steady partners vs no CAI with steady partners

Risk factor	Category	AOR	SE	95% confidence interval		*P*-value
				Lower	Upper	
**Serostatus communication**	Unsuccessful	Ref				
	Successful	2.72	3.66	2.72	3.66	<0.001
**Age**						
	Continuous	0.99	<0.01	0.98	0.99	<0.001
**Outness**	Out to less than half	Ref				
	Out to majority	1.16	0.09	1.00	1.36	0.054
**Had HIV test in last 12 months and results known**	Yes	Ref				
	No	1.26	0.10	1.09	1.46	0.002
Constant		0.50	0.08	0.37	0.67	<0.001
City	Variance (Constant)	0.03	0.02	0.01	0.11	

LR test vs. logistic regression: chibar2(01) = 9.17 Prob. ≥ chibar2 < 0.0012.

*Notes:* Adjusted Odds Ratio (AOR); Standard Error (SE)

### Age

**Fig. 1:**
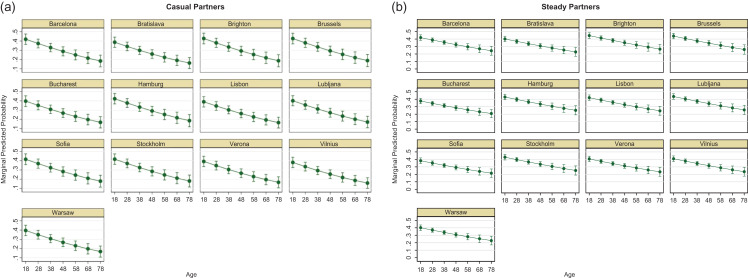
Marginal predicted probabilities of CAI in casual and steady partners, by age group and city.

Figure 1 represents the estimated risk of CAI in respondents who have casual (a) and steady (b) partners by (continuous) age for each of the study cities. The two sets of graphs within Fig. 1 are not directly comparable because they are based on two different models incorporating different underlying theories on behaviours and risk. However, both sets show that overall young MSM are more likely to report higher levels of CAI compared to older MSM and the levels of CAI varies across cities. For instance Brighton has the largest estimated probabilities of CAI in casual partners: at age 18 years (years) *P* = 0.43 and this drops to *P* = 0.19 for older (78 yrs) MSM; Vilnius had the lowest probabilities and estimates ranged from *P* = 0.38 (18 years) to *P* = 0.16 (78 years). For steady partners, again Brighton has the largest estimated probabilities of *P* = 0.43 (18 years) and *P* = 0.26 (78 years); Bucharest had the lowest probabilities ranging from *P* = 0.35 (18 years) to *P* = 0.20 (78 years). Amongst MSM with steady partners, Barcelona, Brighton, Brussels, Hamburg, Lisbon, Ljubljana and Stockholm can all be grouped together as cities with consistently higher probabilities of CAI at each age; similarly Barcelona, Brighton, Brussels, Hamburg, Sofia and Stockholm all had higher probabilities at each age for CAI in casual partners compared to the other study sites.

## Discussion

### Main finding of this study

A number of factors were associated with increased likelihood of CAI between MSM with casual partners including being ‘out’ to a majority, knowing one’s own HIV status, and using substances. Reductions in the likelihood of CAI were associated with being older, as well as successful sero-communication, and not having had a recent HIV test. Being older may be related to having experienced more intensive condom promotion and having witnessed the severe consequences of historically untreatable HIV infection. In terms of successful sero-communication: with casual partners sero-communication may be a surrogate for HIV-related concerns and higher intentions of self-protection, while sero-communication with steady partners may serve to confirm HIV sero-concordance and successful serosorting and to allow more ‘intimacy’ by practising CAI.

For those with at least one casual partner, having sex with a female and being a migrant were not associated with the likelihood of CAI. Similarly for MSM with at least one steady partner, the likelihood of CAI was positively associated with successful sero-communication and not having had a recent HIV test within the last 12 months; it was also negatively associated with increasing age. Interestingly, regardless of partner type, our analysis indicated a downward trend in the probability of CAI with increasing age. The gradual declining trend, and smaller 95% confidence intervals at the margins, indicated that relationships amongst steady partners are more stable whilst casual partners are more variable. These data suggest that regardless of partner type, prevention strategies may benefit from disproportionately targeting younger MSM.

### What is already known on this topic

Previous studies have identified associations between CAI between MSM and relationship status.^4,9,24–26^ Concurring with our own findings, prior studies have also found significant associations between CAI and age with younger MSM seemingly more likely to engage in CAI with steady partners.^24^ In our study this was also the case although irrespective of partner type.

Of potential relevance to our analysis, a recent study from Australia has shown that a rapid increase in pre-exposure prophylaxis (PrEP) use by gay and bisexual men in Melbourne and Sydney was accompanied by an equally rapid decrease in consistent condom use with casual partners.^13^ Future studies may therefore wish to consider the importance of understanding the complex dynamics of partner type/relationship status for the prevention of other STIs as well as considering how CAI behavioural stratification could be used to determine who might benefit from tailored health promotion interventions including HIV PrEP.

### What this study adds

Understandings of how partner type or relationship status may shape sexual behaviour such as CAI amongst MSM in European cities may help to play an important role in the development of culturally appropriate HIV/STI prevention and risk-reduction efforts targeting at-risk MSM. Our findings indicate the need for further investigation on how partner type and other partnership characteristics and dynamics may influence CAI and HIV and/or STI transmission amongst MSM.

### Limitations of this study

Due to the cross-sectional nature of the study design, no causality or temporality between the associations examined can be inferred. An important limitation relates to the sampling methodology. TLS and RDS methods are considered quasi-probabilistic approaches, targeting MSM through their attendance in gay venues (TLS) or via social networks (RDS). This means that such approaches are subject to specific shortcomings such as the possible over- or under-representation of potential MSM sub-samples.^27^ However, TLS and RDS do nevertheless still represent one of the main and current approaches for recruiting most at-risk populations to bio-behavioural surveys.^28^ Survey data can of course be subject to specific biases related to the fact that some data were self-reported (excluding the data on HIV status when based on laboratory testing) limiting generalisability. This implies recall and social desirability bias given behaviours such as CAI were explored. The questionnaire has however been designed to overcome these potential biases, for instance through the active involvement of local gay NGOs in each site.^29^ It is also possible that although we provided descriptions of different partner types in the survey, variations regarding the interpretation of what constitutes a ‘steady’ versus a ‘non-steady/casual’ partner might not be uniform across study participants (e.g. see^7^).

Finally, as an EC co-funded project, the Sialon II project was designed to include cities from countries with different social and cultural contexts. As in many such EC-funded projects, cities were selected on the basis of previous research and collaboration networks and on the basis of pragmatic financial/organisational issues; therefore, some key cities with sizable gay populations have not been covered by the survey.

Despite the above limitations however, our analysis provides important information regarding the association between CAI and partnership characteristics amongst MSM in 13 European cities.
